# A Multi-Color Flow Cytometric Assay for Quantifying Dinutuximab Binding to Neuroblastoma Cells in Tumor, Bone Marrow, and Blood

**DOI:** 10.3390/jcm12196223

**Published:** 2023-09-27

**Authors:** Michelle E. Keyel, Kathryn L. Furr, Min H. Kang, C. Patrick Reynolds

**Affiliations:** 1 Department of Pediatrics, School of Medicine, Texas Tech University Health Sciences Center, Lubbock, TX 79430, USA; michelle.keyel@ttuhsc.edu (M.E.K.); kathryn.furr@ttuhsc.edu (K.L.F.); 2 Departments of Pediatrics and Cell Biology and Biochemistry, School of Medicine, Texas Tech University Health Sciences Center, Lubbock, TX 79430, USA; min.kang@ttuhsc.edu; 3 Department of Pediatrics, Internal Medicine, Cell Biology and Biochemistry, School of Medicine, Texas Tech University Health Sciences Center, Lubbock, TX 79430, USA

**Keywords:** neuroblastoma, GD2, dinutuximab, flow cytometry

## Abstract

GD2, a disialoganglioside, is present on the surface of most neuroblastomas, as well as on some other cancers, such as melanoma and osteogenic sarcoma. The anti-GD2 antibody ch14.18 (dinutuximab) has an FDA-registered indication for use as maintenance therapy for high-risk neuroblastoma with cytokines and 13-cis-retinoic acid after myeloablative therapy. Recent studies using immunohistochemistry of tumor or tumor cells in marrow have shown that some neuroblastomas are negative for GD2. Dinutuximab and other anti-GD2 antibodies are increasingly used in combination with cytotoxic chemotherapy for treating relapsed neuroblastoma, so it is important to be able to identify patients with tumor cells with low GD2 expression, as such patients may experience toxicity but not benefit from the antibody therapy. As the most common clinical samples available for relapsed neuroblastoma are bone marrow aspirates, we developed a method to quantify dinutuximab binding density and the frequency of neuroblastoma cells positive for the antibody in bone marrow aspirates. Here, we describe a multi-color flow cytometry assay that employs non-GD2 antibodies to identify neuroblastoma cells in a mixed population (tumor, bone marrow, or blood) and an anti-GD2 antibody to quantify both the frequency and density of GD2 expression on neuroblastoma cells.

## 1. Introduction

Neuroblastoma is a pediatric cancer of the sympathetic nervous system that is frequently found to express high levels of the disialoganglioside GD2 [1]. As a disialoganglioside, GD2 includes a fatty acid core, two sialic acid residues, and three monosaccharides [2]. GD2 was formerly thought to be present on all neuroblastomas, and, as such, was used as a target for anti-neuroblastoma therapy. Several antibodies against GD2 have been employed for therapy of neuroblastoma, including the chimeric antibody ch14.18 (dinutuximab) [3,4,5], dinutuximab beta [6,7,8], the murine antibody 3F8 [4,9,10,11], a humanized 3F8 (naxitamab) [12,13,14], and a humanized version of ch14.18 [15,16,17,18]. Dinutuximab (ch14.18) and dinutuximab beta are mouse–human chimeric antibodies with registered indications for use as post-consolidation therapy for children with high-risk neuroblastoma [3,5,6,7] that are also used in combination with chemotherapy for treating progressive disease [19]. Naxitamab has a registered indication for treating progressive disease neuroblastoma [20]. CAR-T cells have also been developed against GD2 and have recently shown some clinical activity [21,22].

Recent evidence suggests that as many as 12% of neuroblastomas may be GD2-negative, as determined via immunohistochemical staining of tumor in bone marrow on slides [23]. Another group reported that the percentage of low-GD2 cells in neuroblastoma tumors increased in relapsed patients following anti-GD2 antibody therapy [24]. Our laboratory has also observed low-to-negative GD2 expression on patient-derived neuroblastoma cell lines [25,26].

Antibody therapy that targets GD2 relies on the presence of the GD2 antigen on neuroblastoma cells so that antibody binding can trigger antibody dependent cellular cytotoxicity (ADCC) or complement dependent cytotoxicity (CDC) in patients [3,18,27]. Natural killer cells, macrophages, neutrophils, and complement have all been shown to be involved in the anti-tumor response triggered by anti-GD2 antibodies [28,29,30,31]. However, if GD2 is not present on the surface of neuroblastoma cells, antibodies, such as dinutuximab, have no target to bind to, and therefore will not result in anti-tumor activity. Given the evidence that some neuroblastomas are GD2-negative or contain portions of the tumor population that lack GD2, it is likely that some neuroblastoma patients have at least part of a tumor population that is unable to be targeted with dinutuximab.

Currently, GD2 expression is not routinely evaluated in neuroblastoma patients prior to therapy either at diagnosis or relapse. Therefore, patients with low or negative GD2 expression will be treated with antibody and experience toxicities, including acute pain and hypotension, without likely clinical benefit [32]. With increasing use of dinutuximab, both as maintenance therapy after completion of cytotoxic therapy and also in direct combination with salvage chemotherapy, there is increasing selective pressure for antigen negativity. Tumor biopsies at time of progressive disease are not a part of routine practice, but progressive disease neuroblastoma patients commonly have tumor metastases in the bone marrow and bone marrow aspirates are frequently obtained at diagnosis, at relapse, and to monitor disease progression. Hence, bone marrow aspirates are readily available clinical samples for assessing GD2 with flow cytometric assays.

Several assays have also been proposed using flow cytometry or other methods which rely on the presence of GD2 in addition to other markers on tumor cells to identify neuroblastoma cells in a patient sample [33,34,35]. However, if GD2 is low or not present on the cells, these assays will fail in their assessment of neuroblastoma in a patient sample. Here, we describe a multi-color flow cytometry assay that can detect neuroblastoma cells in patient samples, including in blood, tumor, and bone marrow, and also enables gating on the neuroblastoma cells in a mixed population to quantify both the frequency and the density of dinutuximab binding to tumor cells.

## 2. Materials and Methods

### 2.1. Antibodies

Clinical-grade dinutuximab was generously provided by United Therapeutics (Silver Spring, MD, USA). Monoclonal anti-human neuroblastoma antibodies HSAN1.2 and 459 have been used extensively to detect neuroblastoma cells without binding in human bone marrow or blood [36,37,38]. Dinutuximab was directly conjugated to Alexa Fluor 647 and HSAN was directly conjugated to Alexa Fluor 488 by Pacific Immunology (Ramona, CA, USA). The 459 antibody was directly conjugated to PE-Cy7. Murine anti-human CD45 BV421 clone HI30 monoclonal antibody was purchased from BD Biosciences (San Jose, CA, USA). Human isotype control IgG1 directly conjugated with Alexa Fluor 647 (Dendritics, Lyon, France) and isotype control mouse antibodies for IgG1 and IgM directly conjugated with Alexa Fluor 488 were purchased from R&D Systems (Minneapolis, MN, USA). Anti-human CD276 (B7-H3) Pe-Cy7 clone MIH42 was purchased from Biolegend (San Diego, CA, USA).

### 2.2. Cell Culture

Patient-derived neuroblastoma cell lines (Table 1) from the Alex’s Lemonade Stand Foundation/Children’s Oncology Group repository housed in our lab (www.CCcells.org, accessed on 19 August 2023) were cultured in ether RPMI-1640 supplemented with 10% fetal bovine serum (FBS) or Iscove’s Modified Dulbecco’s Medium (IMDM) (Gibco, Hampton, NH, USA) supplemented with 20% FBS and 1× insulin, transferrin, and selenium (ITS) (Fisher, Hampton, NH, USA). Cell lines were directly established from neuroblastoma patient samples from blood, bone marrow, or tumor received at diagnosis, progressive disease, or progressive disease at post mortem. Details of each of the neuroblastoma cell lines used can be found in Table 1 and at www.CCcells.org. B-cell lymphoma cell lines, Toledo [39] (ATCC CRL-2631) or Farage [40] (ATCC CRL-2630), were employed as a negative control. Cells were established and cultured at 2%, 5%, or 20% O_2_, 5% CO_2_, at 37 °C; the oxygen concentration used for each cell line is listed in Table 1. Oxygen concentrations reflect the culture conditions the patient sample was incubated at when it was established as a cell line (typical 20% O_2_, bone marrow hypoxia = 5% O_2_, and tumor hypoxia = 2% O_2)_. In some cases, a cell line is only established at one oxygen concentration from a single patient sample. The cell lines used were not switched from the oxygen concentration they had been established in. Cell lines were grown to 70% confluence, harvested with Pucks saline A + EDTA on the day of the assay, and centrifuged at 450× *g* for 5 min. All cell lines were verified by short tandem repeat (STR) assay to match the patient of origin using the GenePrint 10 STR system (10 loci + amelogenin) from Promega (Madison, WI, USA). Cell lines were verified to be mycoplasma-free by testing with the MycoAlert^TM^ Mycoplasma Detection Kit (Lonza, Basel, Switzerland). Neuroblastoma cell lines were confirmed to be neuroblastoma as they were highly positive by RT-PCR for tyrosine hydroxylase mRNA.

### 2.3. Seeding of Neuroblastoma Cell Lines into Human Blood

Human whole blood was purchased from ZenBio (Research Triangle Park, Durham, NC, USA), diluted in PBS, laid over LSM lymphocyte separation media (MPBio, Santa Ana, CA, USA), which enriches for viable cells in the mononuclear lymphocyte layer, and centrifuged at 400× *g* for 30 min at room temperature. The isolated mononuclear cells were further treated to lyse red blood cells with 1 mM ammonium bicarbonate + 114 nM ammonium chloride in distilled water (RBC lysis buffer) for five minutes at room temperature. Neuroblastoma cell lines LA-N-1 [43], COG-N-534 [41], and COG-N-519 [41] or COG-N-733h were seeded into human blood at 1.25%, 2.5%, and 5% neuroblastoma to non-neuroblastoma cells at 500,000 cells/tube total. Cells were placed in 5 mL polystyrene round bottom flow cytometry tubes with cell strainer caps (Fisher, Hampton, NH, USA) and washed two times with 1× PBS. Cells were blocked in human Fc block (BD Biosciences, San Jose, CA, USA) in 1% bovine serum albumin (BSA) in 1× PBS for two hours on ice. Cells were washed once in wash buffer (0.5% BSA in 1× PBS) and then stained with the following three-color antibody cocktail: dinutuximab Alexa Fluor 647, HSAN Alexa Fluor 488, and CD45 BV421. Cells were stained for two hours on ice in the dark. Unstained control cells for each cell line and healthy human PBMC alone samples were resuspended in wash buffer for two hours on ice. The appropriate isotype controls were also added to a single population sample for each cell line and PBMC alone and incubated for two hours on ice in the dark. The non-neuroblastoma (lymphoma) cell lines, Toledo or Farage, were used as negative controls and stained with the antibody cocktail. Cells were washed two times in wash buffer and then resuspended in 500 µL per tube of wash buffer. Cells were strained through a 35 µm cell strainer cap before being run immediately on the BD LSRFortessa X-20 (Fortessa) flow cytometer. For detection of Alexa Fluor 647, we employed a 640 nm laser with an emission filter of 670/30 nm. For Alexa Fluor 488, we employed a 488 nm laser with a 530/30 nm filter and 505 LP dichroic. For BV 421, we employed a 405 nm laser with a 450/50 nm filter. For Pe-Cy7, we employed the 561 nm laser with a filter of 780/60 nm and 750 LP dichroic. Ten-thousand events were collected per tube.

### 2.4. Flow Cytometric Evaluation of Neuroblastoma Detection and GD2 Expression

Samples were run on the BD LSRFortessa X-20 using FACSDiva software v9.0 (BD Biosciences, San Jose, CA, USA). Cells were gated from debris using forward and side scatter and then for singlets using first SSC-H vs. SSC-A and then FSC-H vs FSC-A. Neuroblastoma cells were selected by gating on the CD45-negative, HSAN-positive population. GD2 staining (dinutuximab Alexa Fluor 647) was then measured as percentage positive (from the HSAN-positive single-cell population) and median fluorescent intensity (MFI) of the positive population. An unstained control and isotype controls were used for each cell line or patient sample. Single-color controls were also run in each experiment for compensation. Data were analyzed using FlowJo v10.8.1 (Ashland, OR, USA) software. Background fluorescence of the unstained control cell lines was subtracted from the neuroblastoma samples. All MFI values were normalized to the 100% LA-N-1 MFI for each fluorophore by dividing a given MFI by the MFI of LA-N-1 and multiplying by one-hundred. Outlier analysis using the Grubbs’ test was used to remove any outliers from biological replicates (alpha = 0.05) with GraphPad Prism software (https://www.graphpad.com/quickcalcs/Grubbs1.cfm, accessed on 18 August 2023) (Dotmatics, Boston, MA, USA). One-way ANOVA was used to verify that no statistical significance was observed in the MFI variability within a given cell line for both HSAN and GD2 staining.

### 2.5. Preparation of Patient Samples

Fresh tumor, bone marrow, or blood from neuroblastoma patients was collected with informed consent and institutional IRB approval by the Children’s Oncology Group (COG) via the COG biobanking protocol ANBL00B1. Institutions submitted samples to the COG/ALSF Childhood Cancer Repository (www.CCcells.org) located at TTUHSC in Lubbock for establishing patient-derived cell lines and patient-derived xenografts, and for biobanking and distribution of established cell lines and PDXs to other investigators. When sample size permitted, a portion of those specimens were assayed for dinutuximab binding by flow cytometry. Bone marrow and blood samples were prepared the same as healthy donor PBMC as described above. For patient tumor samples, the tumor was minced with scalpels and then strained through a 70 µm nylon cell strainer. The red blood cells were lysed in RBC lysis buffer for five minutes at room temperature. The tumor cells were then washed, counted, and resuspended in 1× PBS.

### 2.6. Staining of Neuroblastoma Patient Samples

Mononuclear cells from patient samples were processed and stained the same as described above in the neuroblastoma cell line mixing experiment with PBMC. Cells were stained in a three-color cocktail of dinutuximab Alexa Fluor 647, HSAN Alexa Fluor 488, and CD45 BV421 or dinutuximab Alexa Fluor647, 459 Pe-Cy7, and CD45 BV421. LA-N-1 was used as a positive control neuroblastoma cell line. The non-neuroblastoma cell lines of Toledo or Farage were used as negative controls. Samples were run on the Fortessa and 100,000 events were collected per tube. Data were analyzed in FlowJo v10.8.1 software. Live cells and singlets were selected using FSC and SSC as described above. The CD45^−^/HSAN^+^ or CD45^−^/459^+^ cells were selected as neuroblastoma cells. From this population, GD2 staining was determined as percentage positive in the neuroblastoma population and also determined was the median fluorescence intensity (MFI). Any background fluorescence from unstained controls was subtracted from the sample fluorescence and MFI values were normalized as percentage of the LA-N-1 positive control.

## 3. Results

### 3.1. Evaluation of Surface Markers on Neuroblastoma Cell Lines

**Figure 1:**
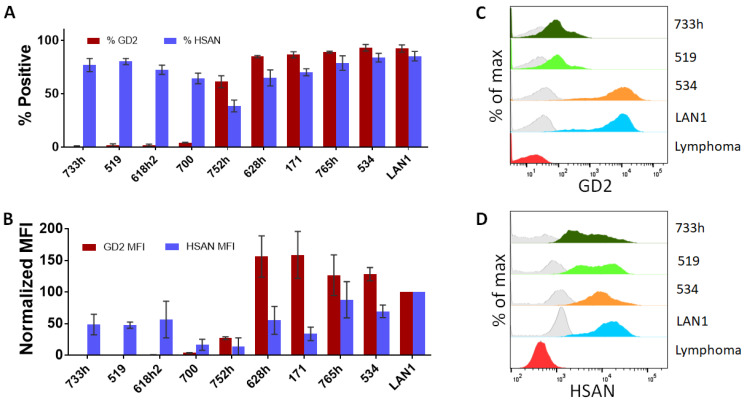
Surface expression of neuroblastoma antigens on neuroblastoma cell lines. (**A**) Ten cell lines were stained for GD2 and HSAN. Positive staining was determined using flow cytometry as percentage positive or (**B**) median fluorescence intensity (MFI) for either HSAN or GD2. MFI for GD2 and HSAN was normalized to LA-N-1, which was set to 100 (n = 3). (**C**) Representative histograms showing the staining for GD2 or HSAN (**D**) for one B-cell lymphoma cell line and four neuroblastoma cell lines from the panel of ten are displayed from a single experiment with % of max. Isotype control staining shown in grey transparent curves. Cell line staining shown in filled curves.

We first validated that GD2 expression could be observed and quantified on neuroblastoma patient-derived cell lines (PDCLs) using dinutuximab directly conjugated to Alexa Fluor 647. The specificity of ch14.18 (dinutuximab) for GD2 has been previously validated [44,45,46]. We characterized dinutuximab binding across a panel of ten patient-derived neuroblastoma PDCLs that represented a variety of disease stages and tumor types. Four cell lines were derived from samples taken at diagnosis, three from progressive disease, and three from progressive disease at post mortem (Table 1). In our panel, six cell lines were established from bone marrow samples, three from blood, and one from tumor. Cells grown in typical culture conditions of 20% O_2_ and also bone marrow-level (5% O_2_), and tumor-level (2% O_2_) hypoxia were represented in our panel. In the panel of ten cell lines, six stained positive with dinutuximab and four cell lines had very low or negative dinutuximab staining (Figure 1A,B). However, all ten cell lines stained positive for HSAN, which has previously been reported to bind to neuroblastoma cells but not hematopoietic cells [36,37,38]. Importantly, neuroblastoma cell lines that had very low or were negative for dinutuximab binding still stained positive for HSAN.

In order to demonstrate that our three-color antibody cocktail could successfully identify neuroblastoma cells from a mixed population using flow cytometry, we utilized a subset of four neuroblastoma cell lines from the original panel. This subset included two neuroblastoma cell lines (LA-N-1 and COG-N-534) that were GD2-positive and two that were GD2-low/negative (COG-N-733h and COG-N-519). The dinutuximab binding profile of GD2 was compared to the isotype controls and a B-cell lymphoma negative control (Figure 1C). The population staining positive with dinutuximab, designated as the GD2-positive population, was analyzed as the percentage GD2^+^ population from the total population of singlet cells and indicated the prevalence of GD2 surface expression being bound by dinutuximab. Heterogeneity of GD2 expression within the population was detected, indicating that, even in an STR verified neuroblastoma cell line, not all cells have detectable antigen on the surface at all times. Furthermore, two neuroblastoma cell lines, COG-N-519 and COG-N-733h, were found to lack a population that stained positive for GD2 surface expression, indicating that not all neuroblastoma cell lines are GD2-positive (Figure 1A,C) while all four neuroblastoma cell lines had detectable levels of HSAN on their surface (Figure 1D).

### 3.2. Validation of Detection of Neuroblastoma Cells from a Mixed Population

**Figure 2:**
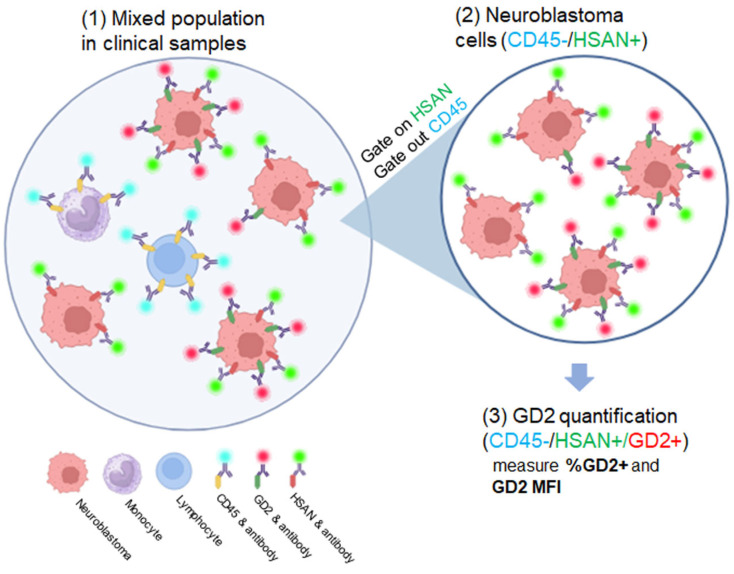
Gating strategy for three-color antibody cocktail. After selection of singlet cells, neuroblastoma cells were gated based on CD45^−^/HSAN^+^ staining in the three-color assay and then analyzed for GD2 staining. Figure created with BioRender.

Based on these results, we designed an assay strategy to gate neuroblastoma cells from a mixed population of cells in clinical samples by identifying neuroblastoma cells using positive HSAN staining and then quantifying GD2 surface levels using dinutuximab Alexa Fluor 647 (Figure 2). CD45 was used as a negative selection antibody, as CD45 is a marker found on leukocytes, but not neuroblastoma [33,35,47,48,49].

**Figure 3:**
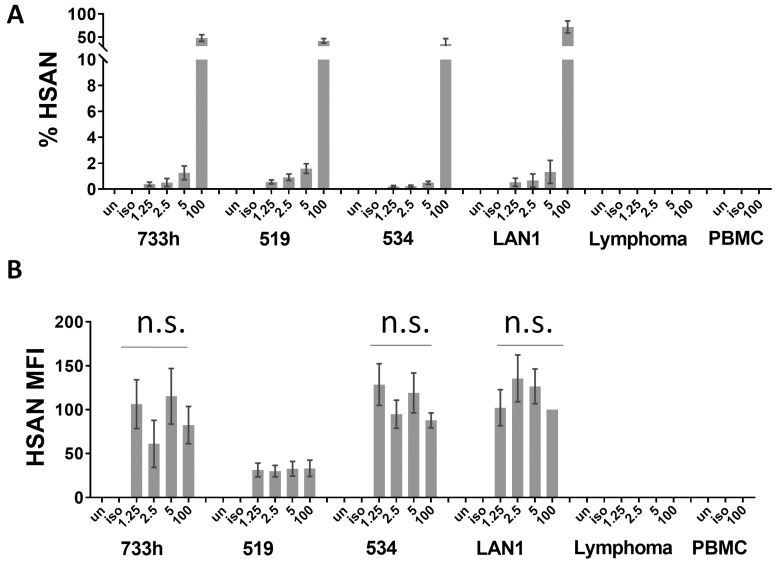
Identification of neuroblastoma cells using three-color flow cytometry. (**A**) LAN-1, COG-N-534, COG-N-519, or COG-N-733h neuroblastoma cell lines were analyzed as pure populations or mixed with PBMC at 1.25%, 2.5%, or 5% neuroblastoma to PBMC. The neuroblastoma was identified from each sample via flow cytometry using the three-color antibody cocktail. Percent of HSAN^+^ cells from the 3-color assay is recorded. Data are displayed as % of the neuroblastoma cells detected from the CD45^−^ population. Each bar is mean percentage from three–four replicates. Error bars are SEM. (**B**) The median fluorescence intensity (MFI) of HSAN is recorded from the neuroblastoma cells detected from the CD45^−^ population. All MFIs were normalized to the 100% LA-N-1 population, which was set to 100. Each bar is the mean percentage from three–four replicates. Error bars are SEM. Variation in MFI within each cell line was determined to be non-significant.

Neuroblastoma cells were seeded into healthy donor human peripheral blood mononuclear cells (PBMC). Singlet cells were first selected, and then CD45 was used as a negative selection gate. Neuroblastoma cells were then identified from the CD45^−-^ population using HSAN as a positive selection marker. This CD45^−^/HSAN^+^ population was designated as the neuroblastoma population. As anticipated, the negative control B-cell lymphoma and the PBMC-only control had minimal background (Figure 3).

In order to determine the sensitivity of our assay to detect neuroblastoma cells, we created serial dilutions of 1.25%, 2.5%, and 5% neuroblastoma cells in PBMC and detected the CD45^−^/HSAN^+^ population in each cell mixture (Figure 3A). We found that, with our three-color antibody staining cocktail, we were able to identify the neuroblastoma cells of each neuroblastoma cell line from the PBMC. The percentage of neuroblastoma cells detected in each mixture increased with each of our increasing dilutions. Importantly, we did not detect a higher percentage of neuroblastoma cells in any of our mixtures than what was actually added, indicating that our assay does not lend itself toward false positives with detection of neuroblastoma cells. Furthermore, the density of HSAN binding on the neuroblastoma cells was measured as MFI (Figure 3B). The MFI did not significantly vary between different samples of the same cell line, once again indicating that the assay can accurately identify the neuroblastoma cells combined with hematopoietic cells at a variety of concentrations with exclusion of non-neuroblastoma cells. Our assay was not able to distinguish the negative control B-cell lymphoma cell line from the PBMC using HSAN. In the negative control, no false positive signals were detected.

### 3.3. Quantification of GD2 Surface Expression from Neuroblastoma Cells in a Mixed Population

**Figure 4:**
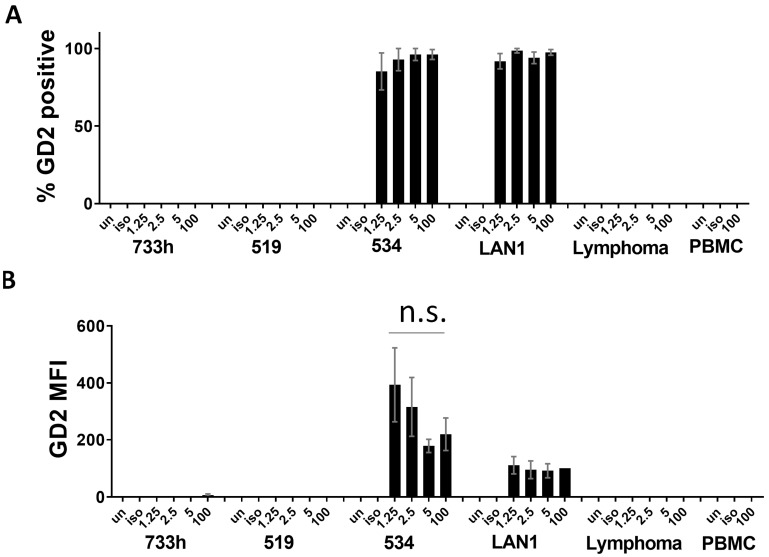
Quantification of GD2 surface expression from mixed neuroblastoma populations. (**A**) GD2 surface expression was measured from the neuroblastoma cells identified from the 100%, 5%, 2.5%, or 1.25% neuroblastoma mixed with PBMC as percentage GD2-positive or GD2 MFI. (**B**) MFI was normalized to the MFI of 100% LA-N-1, which was set to 100. Each bar is the mean from three–four replicates. Error bars are SEM. Variation in MFI within each cell line was determined to be non-significant.

After successfully identifying neuroblastoma cells from the cell dilutions in PBMC, we quantified the GD2 surface expression of the neuroblastoma cells, as indicated by dinutuximab binding. The percentage of neuroblastoma cells detected from each dilution of LA-N-1 into PBMC that were GD2-positive was tested for each dilution. Importantly, the percentage of neuroblastoma cells that was GD2-positive was similar in each mixed population to the percentage of GD2-positive cells seen in the 100% LA-N-1 population (Figure 4A). The percentage of GD2-positive cells was also very similar for each dilution of COG-N-534 and matched what was seen in the pure neuroblastoma cell population. These data indicate that we could accurately assess the prevalence of GD2 surface expression within a given sample, even when neuroblastoma mixed with hematopoietic cell populations at different cell ratios. Furthermore, we could quantify the antigen density of GD2 using the MFI of dinutuximab from the identified neuroblastoma population. For both LA-N-1 and COG-N-534, at all mixing ratios, we were able to identify GD2 antigen densities that did not significantly differ from the GD2 MFI of the 100% neuroblastoma populations (Figure 4B). Thus, we were able to accurately describe the GD2 density of a given neuroblastoma population in a mixed population using our three-color flow assay. GD2 surface expression was not detected in COG-N-519 or COG-N-733h, which are low-GD2-expressing models (Figure 1).

### 3.4. Identification of Neuroblastoma Cell Lines from Patient Blood, Bone Marrow, and Tumor Samples

**Figure 5:**
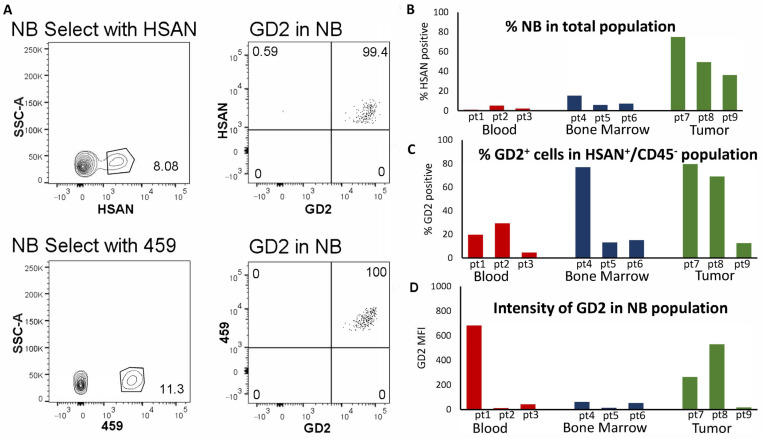
GD2 frequency and density within the neuroblastoma population from patient bone marrow, blood, and tumor samples. (**A**) Neuroblastoma cells were identified from a patient bone marrow sample as CD45^−^/HSAN^+^ and then the percentage of GD2 staining was quantified in the neuroblastoma population (top panels). The same patient BM sample was also stained with a cocktail of anti-CD45 BV421, anti-GD2 Alexa Fluor 647 (labeled dinutuximab), and the anti-neuroblastoma antibody 459 Alexa Fluor Pe-Cy7, and the neuroblastoma cells were identified using the CD45^−^/459^+^ gate and then the frequency of GD2 surface expression from this population was quantified (bottom panels). (**B**) Representative data from three blood, three bone marrow, and three tumor patient samples are shown. Each sample is from a different patient (pt1–9). The neuroblastoma population was selected using the CD45^−^/HSAN^+^ gate from the 3-color antibody cocktail and displayed as percentage of neuroblastoma cells from the total population. The percentage positive (**C**) for dinutuximab staining and the MFI (**D**) of the dinutuximab staining was then quantified from the neuroblastoma population.

The goal for this assay is to detect neuroblastoma cells in a mixed population from human samples, such as bone marrow metastases, so that GD2 prevalence and the density can be quantified. We next determined GD2 expression in clinical samples using the assay developed. After selecting for singlets, CD45^−^/HSAN^+^ cells were selected to identify neuroblastoma cells (Figure 5A, top panels) within a patient sample population from bone marrow via flow cytometry. From the neuroblastoma population, the frequency (% positive) of GD2-positive cells and the density of GD2 (MFI) were determined. We further validated the specificity and sensitivity of HSAN for positively gating on neuroblastoma cells in marrow by also using another non-GD2 neuroblastoma antibody (459) [37,38] that does not bind to normal blood or bone marrow. The 459 antibody was directly conjugated with Pe-Cy7 and used in combination with anti-CD45 and dinutuximab to yield very similar results as seen using CD45, HSAN, and dinutuximab in the same patient sample (Figure 5A, bottom panels). The gating strategy used for the clinical sample can be seen in greater detail in Supplementary Figure S1.

As seen in the top panel of Figure 5B, neuroblastoma cells were detected from patient blood, bone marrow, and tumor samples using the three-color antibody cocktail with HSAN. The highest percentage of neuroblastoma detected was from solid tumor samples, as expected. The frequency of GD2 within the neuroblastoma population, as demonstrated by dinutuximab binding, varied among samples, with some samples containing a low percentage of GD2-positive cells (Figure 5C). The density of GD2, as indicated by dinutuximab median fluorescence intensity (MFI), was also quantified for each patient sample (Figure 5D). The density of GD2 was found to be low in some samples, even though there was high frequency of GD2-positive cells.

## 4. Discussion

Dinutuximab, either together with cytokines as post-consolidation therapy after completion of cytotoxic therapy for patients in first response, or added to other chemotherapeutic agents for treating progressive disease, is used as part of the standard therapy for high-risk neuroblastoma patients [50]. Anti-neuroblastoma activity of dinutuximab relies on the presence of its target, GD2, on the surface of neuroblastoma cells. It has recently been shown, by us and others, that not all neuroblastoma tumors are GD2-positive [23,25]. Neuroblastoma patients with tumors not expressing the GD2 antigen, with a low density of GD2, or with a low frequency of GD2-positive cells may not benefit from anti-GD2 therapy. In this study, we have demonstrated the ability to identify neuroblastoma cells using non-GD2 markers to enable downstream analysis of GD2 frequency and density. We demonstrated that, using our validated three-color antibody cocktail, we could identify neuroblastoma cells that were mixed into healthy donor PBMC or neuroblastoma that were present in patient bone marrow, blood, or tumor, allowing for analysis of GD2 antigen expression only on neuroblastoma cells. This simplified yet accurate assay maximizes its accessibility to other laboratories, enabling potential use as a biomarker for analysis of clinical samples.

Using multi-color flow cytometry, we have demonstrated that neuroblastoma cells can be identified from patient blood, bone marrow, or tumor samples independently of GD2 antigen expression and that GD2 frequency and density can then be quantified within the neuroblastoma population. The assay described here has high sensitivity that enables us to detect a minor population from patient blood or bone marrow, through the use of both negative and positive selection. Although some previous studies have shown greater sensitivity with their multicolor flow cytometry assays, they relied on GD2 expression on their samples [35,48,49]. We have shown this type of assay cannot be used to identify neuroblastoma in a mixed population if the goal of the assay is to identify GD2-low or -negative tumors. Furthermore, some of their analyses involved moving the selection gates based on the variability of staining specific to each of the test neuroblastoma cell lines. We did not adjust gates within any cell line during acquisition or analysis in a given experiment. This better represents the gating strategy needed to identify an unknown neuroblastoma population in an actual patient sample, when the exact staining profile of the tumor is unknown. Gates need to be set in a manner that captures a variety of staining profiles found on neuroblastoma cells, without compromising the integrity of the assay or drastically increasing false positives.

Some other studies also used other neuroblastoma markers to select for neuroblastoma cells in their flow cytometry assays, such as B7-H3 [48]. However, we have observed that B7-H3 is not expressed on all neuroblastoma cells, with cell lines such as COG-N-733h being B7-H3-low/negative (Supplementary Figure S2). Therefore, the addition of this marker to our panel would not necessarily enhance our ability to detect all neuroblastoma from a mixed population sample. However, our three-color assay is amenable to the addition of other markers, such as on the Pe-Cy7 channel, which we used for 459. A cell viability dye, such as one with a 405 nm excitation, can also be added to our assay in combination with our antibodies to gate out the small number of dead cells in patient samples that would not be eliminated in the LSM lymphocyte separation media used during sample preparation. However, in exploring this approach we did not find that use of a cell viability dye altered assay results. The antibody cocktail and gating strategy described here can be applied to cell sorting as well, enabling the capture of a GD2low or high population of cells from a patient sample for future analysis. The intentionally simplified format of the assay makes it easy to use across multiple flow cytometry platforms and with easily accessible data analysis tools.

This assay can be used to identify patients with tumors having low GD2 expression at diagnosis or relapse, enabling determination of the impact of selective pressures during therapy on GD2 expression in patient tumor cells. It is possible patients that are GD2-negative/-extremely low in our assay have an altered form of GD2 or GD2 that is not localized to the cell surface. However, since we are using a labeled form of the clinically available dinutuximab to identify GD2 in patient samples, the inability of our assay to detect GD2 would also indicate that the therapeutic dinutuximab antibody would be unable to bind GD2 for any therapeutic benefit within these patients.

In collaboration with the Children’s Oncology Group, we are employing this assay to determine if low levels of dinutuximab binding are associated with a low response to chemoimmunotherapy that includes dinutuximab. Demonstration that this assay can identify patients unlikely to respond to anti-GD2 therapy would enable use of this assay to aid in interpreting results from clinical trials of anti-GD2 therapy and potentially to guide selection of therapy for neuroblastoma patients.

## Figures and Tables

**Table 1:** Characteristics of neuroblastoma cell lines used for the current study.

Cell Lines	Oxygen Condition	Phase of Therapy	Sample Type
COG-N-765h	5%	DX	BM
COG-N-752h	5%	PD	BM
**COG-N-733h**	**5%**	**DX**	**BM**
COG-N-700	20%	DX	tumor
COG-N-628h [41]	5%	DX	BM
COG-N-618h2	2%	PD	BM
**COG-N-534 [41]**	**20%**	**PD-PM**	**blood**
**COG-N-519 [41]**	**20%**	**PD-PM**	**blood**
CHLA-171 [42]	20%	PD-PM	blood
**LA-N-1 [43]**	**20%**	**PD**	**BM**

Table 1. Characteristics of the panel of 10 neuroblastoma cell lines. Patient-derived neuroblastoma cell lines were established from bone marrow (BM), blood, or tumor samples received from diagnosis (DX), progressive disease (PD), or progressive disease at post mortem (PD-PM). Neuroblastoma cell lines were grown in 2%, 5%, or 20% O_2_. Cell line names in bold were chosen for our panel of four neuroblastoma cell lines.

## Data Availability

All data in this manuscript are contained in the figures of the manuscript.

## Supplementary Materials

The following supporting information can be downloaded at: https://www.mdpi.com/article/10.3390/jcm12196223/s1, Figure S1: Gating strategy for patient bone marrow using a three-color antibody cocktail; Figure S2: B7-H3 staining profile in neuroblastoma panel.
