# Supplementary material for: A Multi-Color Flow Cytometric Assay for Quantifying Dinutuximab Binding to Neuroblastoma Cells in Tumor, Bone Marrow, and Blood
Source: J Clin Med. 2023 Sep 27;12(19):6223. doi: 10.3390/jcm12196223 (PMC10573805; doi:10.3390/jcm12196223)
Supplement: Supplementary file 1 [file jcm-12-06223-s001.zip › jcm-2594930-supplementary.pdf]

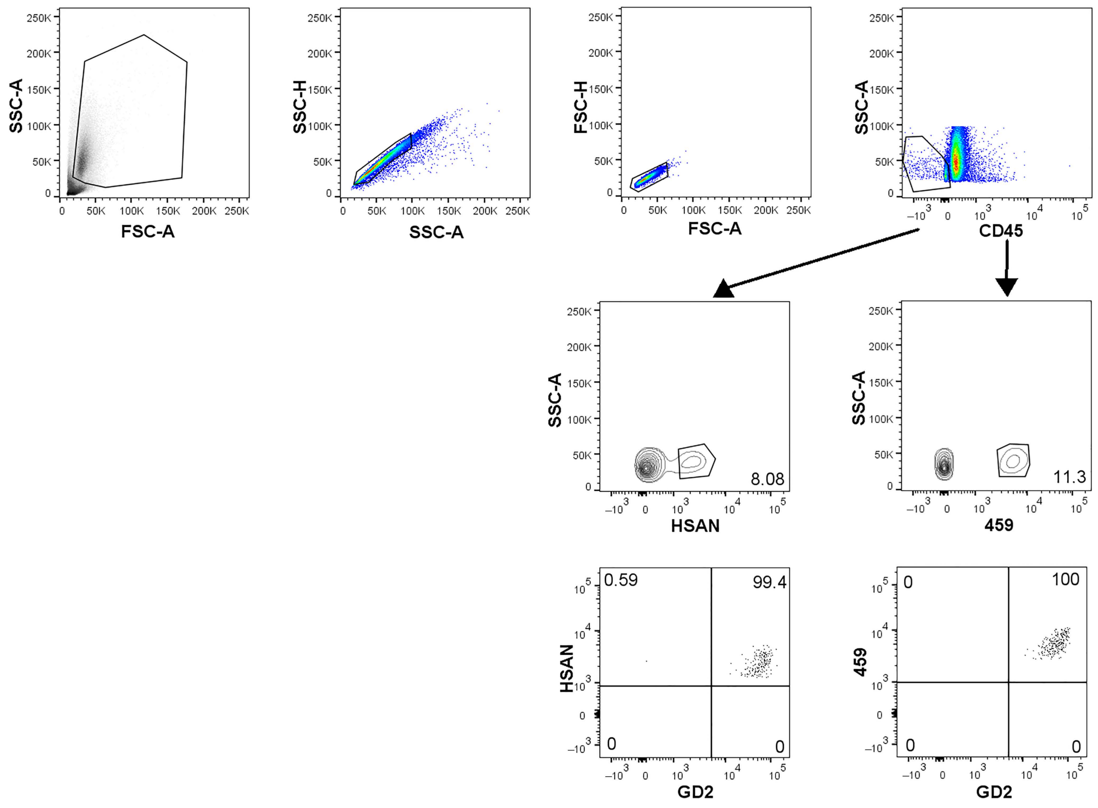

Figure S1. Gating strategy for patient bone marrow using a 3-color antibody cocktail. Cells were selected from debris using forward (FSC) and side (SSC) scatter. Singlets were gated on twice, using both SSC and FSC. The CD45⁻ population was gated on and then from that population, either HSAN⁺ or 459⁺ cells were identified. From each subsequent gate (either CD45⁻/HSAN⁺ or CD45⁻/459⁺) GD2⁺ cells were gated on.

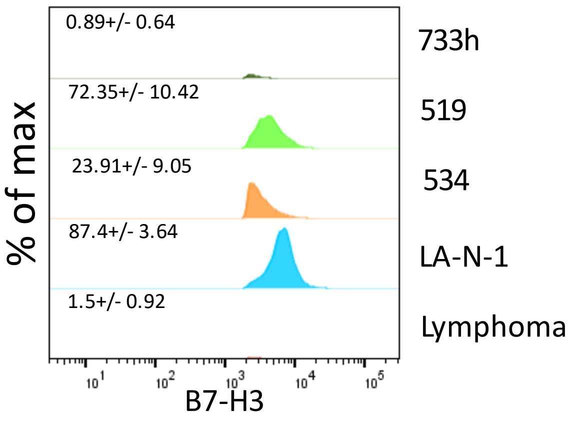

Figure S2. B7-H3 staining profile in neuroblastoma panel. B7-H3 surface staining was assessed on a panel of four neuroblastoma cell lines in addition to the B cell lymphoma negative control. Average % positive from three replicates shown as numbers +/- standard deviation.
